# COPING STYLES AND COGNITIVE FUNCTION IN OLDER NON-HISPANIC BLACK AND WHITE ADULTS

**DOI:** 10.1093/geroni/igac059.124

**Published:** 2022-12-20

**Authors:** Ji Hyun Lee, Ketlyne Sol, Afsara Zaheed, Emily Morris, Lindsey Meister, Jordan Palms, Alexa Martino, Laura Zahodne

**Affiliations:** University of Michigan, Ann Arbor, Michigan, United States; University of Michigan, Ann Arbor, Michigan, United States; University of Michigan, Ann Arbor, Michigan, United States; University of Michigan, Ann Arbor, Michigan, United States; University of Michigan, Ann Arbor, Michigan, United States; University of Michigan, Ann Arbor, Michigan, United States; University of Michigan, Ann Arbor, Michigan, United States; University of Michigan, Ann Arbor, Michigan, United States

## Abstract

Coping styles refer to cognitive and behavioral patterns used to manage the demands of stressors. This study aimed to characterize associations between coping styles and cognitive functioning across non-Hispanic Black and non-Hispanic White older adults. Cross-sectional data comes from the Michigan Cognitive Aging Project (N=457; 53% non-Hispanic Black). Coping styles are measured with COPE inventory. Global cognition was a composite of five cognitive domain factor scores derived from a comprehensive battery. Results show that Black older adults reported more emotion-focused coping than Whites, but there were no differences in problem-focused coping. Less emotion-focused and greater problem-focused coping were each more strongly associated with better global cognition among Black older adults, who showed disproportionately worse cognitive performance in the context of less adaptive coping. Coping style may be a particularly important psychosocial resource for cognitive health among Black older adults who may have less access to compensatory resources than Whites.

